# Betaine Supplementation Causes an Increase in Fatty Acid Oxidation and Carbohydrate Metabolism in Livers of Mice Fed a High-Fat Diet: A Proteomic Analysis

**DOI:** 10.3390/foods11060881

**Published:** 2022-03-19

**Authors:** Caiyun Fan, Haitao Hu, Xiaoyun Huang, Di Su, Feng Huang, Zhao Zhuo, Lun Tan, Yinying Xu, Qingfeng Wang, Kun Hou, Jianbo Cheng

**Affiliations:** College of Animal Science and Technology, Anhui Agricultural University, Hefei 230036, China; fancaiyunnmgbt@163.com (C.F.); huhaitao711@126.com (H.H.); hxyhxy961210@163.com (X.H.); ahausd@163.com (D.S.); f303459358@163.com (F.H.); zhuozhao90@163.com (Z.Z.); tanlun0713@163.com (L.T.); xyy706566358@163.com (Y.X.); wqfyyds1997@163.com (Q.W.); hkljr1998@163.com (K.H.)

**Keywords:** high-fat diet, betaine, murine liver, proteomics

## Abstract

Betaine, a common methyl donor whose methylation is involved in the biosynthesis of carnitine and phospholipids in animals, serves as food and animal feed additive. The present study used liquid chromatography-mass spectrometry (LC-MS) to analyze the liver protein profile of mice on a high fat (HF) diet to investigate the mechanism by which betaine affects hepatic metabolism. Although betaine supplementation had no significant effect on body weight, a total of 103 differentially expressed proteins were identified between HF diet + 1% betaine group (HFB) and HF diet group by LC-MS (fold change > 2, *p* < 0.05). The addition of 1% betaine had a significant enhancement of the expression of enzymes related to fatty acid oxidation metabolism, such as hydroxyacyl-Coenzyme A dehydrogenase (HADHA), enoyl Coenzyme A hydratase 1 (ECHS1) (*p* < 0.05) etc., and the expression of apolipoprotein A-II (APOA2) protein was significantly reduced (*p* < 0.01). Meanwhile, the protein expression of glyceraldehyde-3-phosphate dehydrogenase (GAPDH) and succinate-CoA ligase (SUCLG1) were highly significant (*p* < 0.01). Pathway enrichment using the Kyoto Encyclopedia of Genes and Genomes (KEGG) revealed that the functions of differential proteins involved fatty acid catabolism, carbohydrate metabolism, tricarboxylic acid cycle (TCA) and peroxisome proliferator-activated receptor alpha (PPARα) signaling pathway. Protein–protein interaction (PPI) analysis discovered that acetyl-Coenzyme A acetyltransferase 1 (ACAT1), HADHA and ECHS1 were central hubs of hepatic proteomic changes in the HFB group of mice. Betaine alleviates hepatic lipid accumulation by enhancing fatty acid oxidation and accelerating the TCA cycle and glycolytic process in the liver of mice on an HF diet.

## 1. Introduction

Betaine, a modified amino acid consisting of glycine with three methyl groups, has dipolar zwitterion structure and thus helps to maintain the osmotic equilibrium of cells [1]. Betaine was also a common methyl donor and its methylation was involved in the methionine–homocysteine cycle and in the biosynthesis of both carnitine and phospholipids [2,3], which was widely distributed in plants, animals and microorganisms and had antioxidant, anti-inflammatory and hepatoprotective effects [4]. A large number of studies showed that betaine has been used as a feed additive to regulate meat quality, fat metabolism, etc. in animals and had considerable feeding benefits [5,6]. The liver was the main venue for β-oxidation of fatty acids in mice and is an important organ in the regulation of fat accumulation in the body [7]. In addition, the liver maintains the balance of fat metabolism in the body through the synthesis and secretion of lipoproteins. Therefore, understanding the changes in protein profiles associated with betaine affecting lipid synthesis and metabolism in animal livers is important for gaining insight into betaine and its applications. In recent years, betaine has been extensively studied as a methyl donor with liver-protective effects [8]. Betaine supplementation significantly reduced triglyceride levels in the livers of rats fed an HF diet, but it had no significant effect on the body weights of the animals as compared to those fed a control diet [9]. HF diets supplemented with 1% or 2% betaine significantly reversed S-adenosylmethionine and S-adenosylhomocysteine concentrations in the liver while increased the level of DNA methylation in the liver [10]. Previous research confirmed that the hepatoprotective activity of betaine in HF-induced fatty liver is due to its ability to regulate the sulfur-amino acid metabolism [11]. Betaine supplementation alleviated abnormal adipokine (adiponectin, resistin and leptin) levels in the livers of animals fed an HF diet and attenuated insulin resistance [12]. A study reported that the addition of at least 4 g of betaine per week to diets reduced homocysteine levels in human plasma [13]. Other studies showed that betaine inhibited the synthesis of liver lipids and alleviated endoplasmic reticulum stress and inflammatory responses, thereby reducing the accumulation of liver lipid [14,15,16]. Research showed that supplementation of mice on an HF diet with methyl donors inhibited further accumulation of hepatic fat by activating AMPK-mediated fatty acid utilization [17]. Proteomics provides a rapid and efficient approach to detect protein expression and thus helps us to better analyze the molecular mechanisms which influences liver metabolism. The above studies have demonstrated that betaine can affect the level of DNA methylation and regulate the metabolism of sulfur amino acids in the liver. Nevertheless, there is a shortage of systematic studies on betaine affecting the key pathways and enzymatic activities involved in the metabolism and resynthesis of lipids in the liver. The purpose of this study was to analyze the protein metabolic profile in the liver of mice on an HF diet supplemented with 1% betaine using proteomics. Pathway analysis was also performed in a database to find changes of key pathways and protein expression of enzymes associated with lipid synthesis and metabolism in the liver affected by betaine.

## 2. Materials and Methods

### 2.1. Animals and Diets

Thirty-six 7-week-old male mice weighing 25.83 ± 2 g were randomly divided into four groups of nine mice each. Animals were fed with different diets: control diet (C), control diet supplemented with 1% betaine (CB), HF diet (HF), HF diet supplemented with 1% betaine (HFB). The animals in each treatment group had access to the respective diet and water ad libitum, and the 1% betaine in the CB and HFB group was provided to the mice by the means of drinking water. The details of the diets are shown in Table 1. The experiment lasted for 5 weeks. Water was changed at 08:00 every morning. The animals were weighed once a week, and 24-h food intake per cage was measured once a week, taking spillages into account.

### 2.2. Protein Preparation and Digestion

The liver tissue from each mouse was manually homogenized into powder in liquid nitrogen using a mortar and pestle. Samples with equal weight tissue (200 mg) from each group were pooled into three fractions. The powder was mixed with lysis buffer (8 M urea (Sangon Biotech Co., Ltd., Shanghai, China), 2 M thiourea (Sangon Biotech Co., Ltd., Shanghai, China), 4% CHAPS (Sangon Biotech Co., Ltd., Shanghai, China), 20 mM Tris-base (Sangon Biotech Co., Ltd., Shanghai, China), 30 mM dithiothreitol (Sangon Biotech Co., Ltd., Shanghai, China)) and periodically vortexed in ice for 30 min and then centrifuged at 10,000× *g* for 15 min at 4 °C. Then protein pellets were dissolved with 100 µL of 5 M urea and then mixed with four volumes of 40 mM NH_4_HCO_3_ (Sinopharm Chemical Reagent Co., Ltd., Shanghai, China) solution. The protein concentration was determined using a bicinchoninic acid (BCA) (Beyotime Biotechnology, Shanghai, China) assay. The protein samples were reduced with dithiothreitol solution (final concentration 10 mM) and then alkalized with iodoacetamide (Sinopharm Chemical Reagent Co., Ltd., Shanghai, China) solution (final concentration: 50 mM). Subsequently, protein samples were digested with sequencing-grade modified trypsin (Beyotime Biotechnology, Shanghai, China) at 37 °C overnight (protein:trypsin = 40:1) [18].

### 2.3. Protein Identification

The tryptic peptides were subjected to EASY-nLC1200 (Thermo Fisher Technology (China) Co., Ltd, Shanghai, China) coupled to a Q-Exactive (Thermo Fisher Scientific) system. Before analytical separation, the peptides samples were loaded onto a trap Aqua C18 column with buffer A (Biomiga San Diego, CA, USA). Separation of peptides was performed on an analytical column. The eluted peptides were analyzed using a Q-Exactive mass spectrometer in the positive ion mode and data-dependent mode [19]. The MS data were obtained in full scan mode, with resolution power of 70,000 at 400 *m*/*z*. The top 20 MS/MS were selected from each scan for high energy dissociation analysis.

### 2.4. Data Search

Raw data files were acquired by Xcalibur software, version 2.2 (Thermo Fisher Technology (China) Co., Ltd, Shanghai, China). The raw MS data were compared with data in the UniProt database using PEAKS software, version 7.0 (Bioinformatics Solutions Inc., Waterloo, ON, Canada). PCA analysis was conducted by SIMCA-P+ to identify differences in protein expression between four groups. Cluster analysis of differentially expression of proteins in each group using The R Programming Language, version 4.0 (University of Science and Technology of China). Gene Ontology (GO) annotation analysis of the differentially expressed genes were performed using DAVID, version 6.7 (https://david.ncifcrf.gov/, (accessed on 18 January 2022)). Pathways were enriched and visualized of differentially expressed proteins in four groups by using Kyoto Gene and Genome Encyclopedia (KEGG), (Kyoto University, https://www.kegg.jp/, (accessed on 18 January 2022)). The STRING database (version 10.5, https://string-db.org/, (accessed on 18 January 2022)) was used to establish and detect possible protein–protein interactions (PPI) for the differentially expressed proteins in the HFB group.

Identified proteins were quantified using a label-free strategy with spectral intensities 2. Normalized spectral ion intensities of each sample were analyzed by a one-way ANOVA using SPSS software, version 16.0 (SPSS Inc., Chicago, IL, USA). Differentially expressed proteins in the different treatment groups were considered statistically significant with a *p* value of less than 0.05 and at least a two-fold change.

### 2.5. Western Blot Analysis

Each sample of protein (10 μg) was separated by electrophoresis on an SDS-polyacrylamide gel, then transferred to a polyvinylidene fluoride (PVDF) membrane (Merck Millipore GmbH, Darmstadt, Germany) and the unbound surface of the membrane was sealed with 5% skimmed milk powder for 1 h. Anti-primary antibody (1:1000 dilution, Abcam, Cambridge, MA, USA) was added and incubated overnight at 4 °C. The membrane was then incubated with secondary antibody (1:3000 dilution, Abcam, Cambridge, MA, USA) for 2 h at 25 °C with three times washing. Protein-forming bands were displayed using Novex ECL (Thermo Fisher Scientific Ltd., Rockford, IL, USA). Western blot band results were quantified using Image J software (National Institutes of Health, Bethesda, MD, USA).

## 3. Results

### 3.1. Body Weight and Food Intake

**Figure 1:**
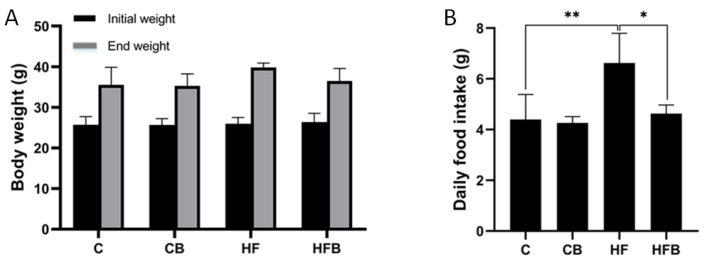
Effect of betaine supplementation on body weight (**A**) and daily food intake (**B**) on mice. Values are mean ± SEM (*n* = 9). * *p* < 0.05, ** *p* < 0.01. C: control, CB: control with 1% betaine, HF: high fat diet, HFB: HF with 1% betaine.

Figure 1A showed the changes in body weights. The body average weight of the mice in the HF group was higher than those in the other groups, but there was no significant difference in the body weights of the other groups at the end of the 5-week period (*p* > 0.05). As shown in Figure 1B, there was a significant difference in daily food intake in the HF group as compared to other groups (*p* < 0.05).

### 3.2. Protein Profiling in Murine Liver

**Figure 2:**
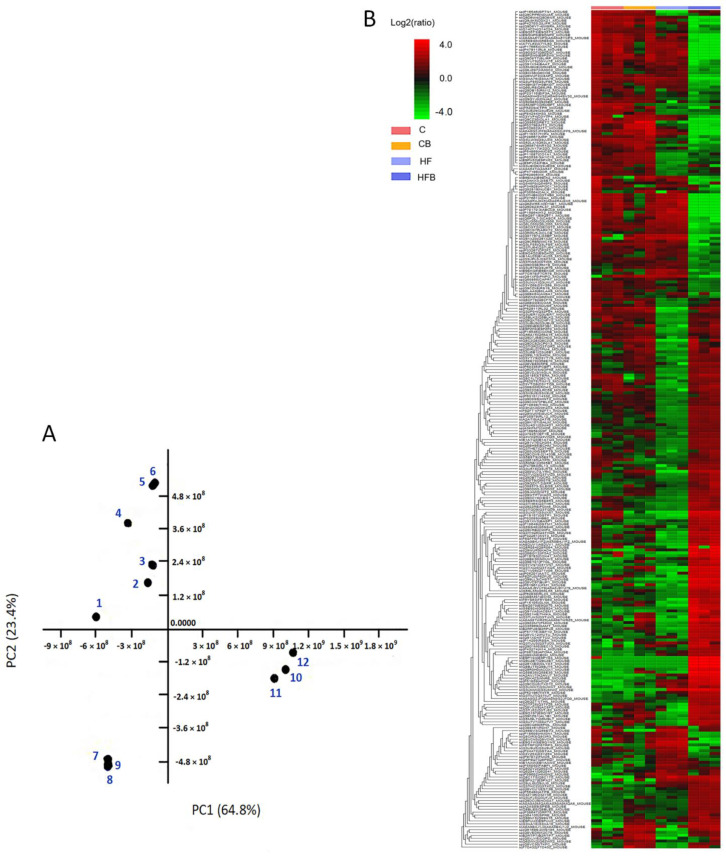
Results of PCA analysis and clustering analysis of liver protein profiles in four treatment groups of mice. (**A**) PCA of non-targeted metabolite profiling in liver using EASY-nLC coupled with Q-Exactive. The points 1, 2 and 3 in the diagram indicate group C; points 4, 5 and 6 indicate group CB; points 7, 8 and 9 indicate HF group and points 10, 11 and 12 indicate group HFB. (**B**) Heat map analysis of differential protein expression in the four groups, red and green indicate high and low concentrations of protein, respectively.

The changes in liver proteins in the different treatment groups of mice can be observed from the separate clustering in principal component analysis (PCA) (Figure 2A). The PCA revealed that 64.8% of all variance in the dataset was accounted for by the first principal component (PC1), and an additional 23.4% was accounted by the second principal component (PC2). The greatest treatment effect was in the HF group (7, 8, 9 spots) followed by the HFB group (10, 11, 12 spots). The protein profiles in the C and CB groups were most similar in heat map, with obvious differences in the protein profiles in the HF and HFB groups (Figure 2B). The results of the PCA were consistent with those of the heat map analysis.

### 3.3. Differences in Protein Regulation in Response to the Betaine Treatment

**Figure 3:**
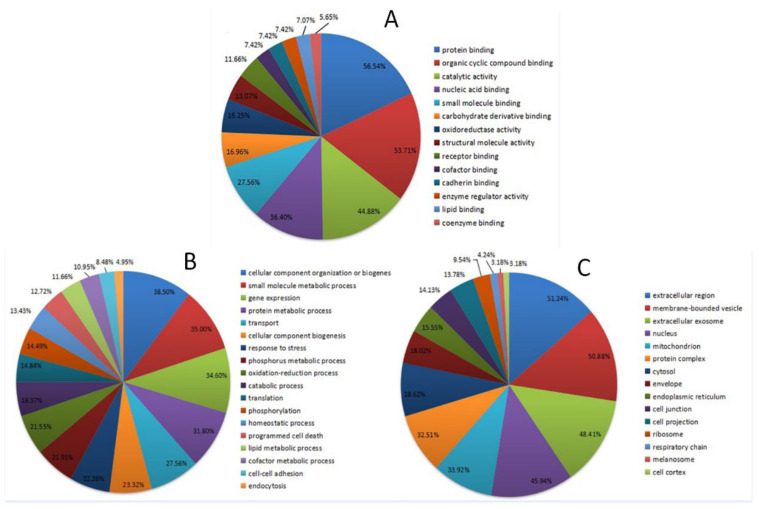
Results of GO analysis of differential proteins in HF group and HFB group. Molecular functions (**A**), biological processes (**B**), cellular functions (**C**).

A nano-LC-MS approach was used to investigate protein profiles in murine liver in the different treatment groups, and 289 proteins were identified in total. Compared with the HF group, 103 proteins were identified as significantly differentially expressed (fold change > 2, *p* < 0.05) in the HFB group, including 71 upregulated and 32 downregulated proteins. The results of the GO analysis were shown in Figure 3. The molecular function analysis showed that 56.54% of the identified proteins were involved in protein blinding, and 7.07% of the proteins were associated with lipid binding processes (Figure 3A). Biological processes that were commonly associated with the HF diet were protein metabolic processes, lipid metabolic processes, catabolic processes and gene expression (Figure 3B). The cellular components identified were extracellular exosomes, mitochondria and endoplasmic reticulum (Figure 3C).

**Figure 4:**
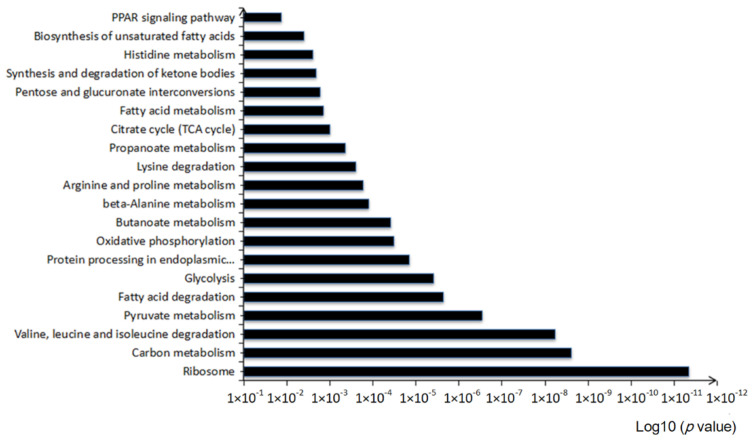
KEGG pathway analysis of differentially expressed proteins in the HF group and HFB group.

In the KEGG pathway analysis, 78 pathways were enriched. The KEGG pathway analysis of the identified proteins showed that these pathways were mostly related to 20 KEGG pathways (*p* < 0.05), including carbon metabolism, fatty acid degradation, the TCA cycle and PPARα signaling pathway (Figure 4).

### 3.4. Differentially Expressed Proteins in the HF and HFB Groups

**Figure 5:**
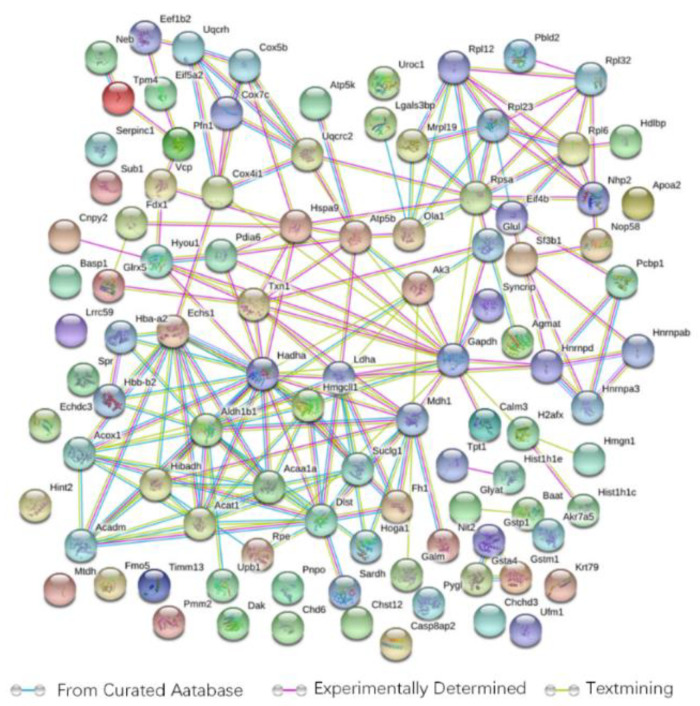
The signaling pathway of betaine affecting the liver protein profile of mice on an HF diet analyzed by the STRING database. Green lines: curated databases, purple lines: experimentally determined, yellow lines: textmining.

The results of the PPI analysis revealed a protein network comprising 99 points (proteins) (Figure 5). When we combined the KEGG pathways and protein association networks, the following hubs were involved in multiple pathways: hydroxyacyl-Coenzyme A dehydrogenase (HADHA), enoyl-coenzyme A hydratase (ECHS1), glyceraldehyde-3-phosphate dehydrogenase (GAPDH), succinyl-CoA ligase subunit alpha (SUCLG1), 3-ketoacyl-CoA thiolase A peroxisomal (ACAA1), acetyl-coenzyme A acetyltransferase (ACAT1), apolipoprotein A2 (APOA2) and acyl-coenzyme A oxidase 1 (ACOX1). In the protein network, ACAT1, HADHA and ECHS1, which served as the central hubs, exhibited more interactions than the other proteins. Interestingly, those three proteins mentioned above were also upregulated in the HFB group and were directly linked to the other changes in the proteins we identified.

**Figure 6:**
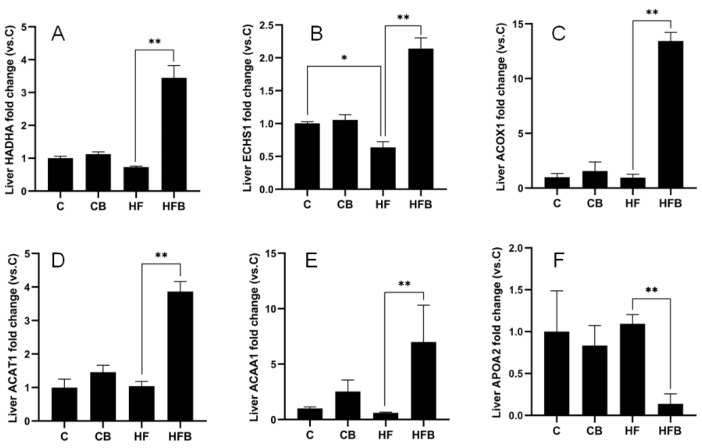
The relative expression HADHA (**A**), ECHS1 (**B**), ACOX1 (**C**), ACAT1 (**D**) and ACAA1 (**E**), APOA2 (**F**), as measured by MS protein profiling. The results are expressed as means ± SEM of the relative peak areas. * *p* < 0.05, ** *p* < 0.01.

**Figure 7:**
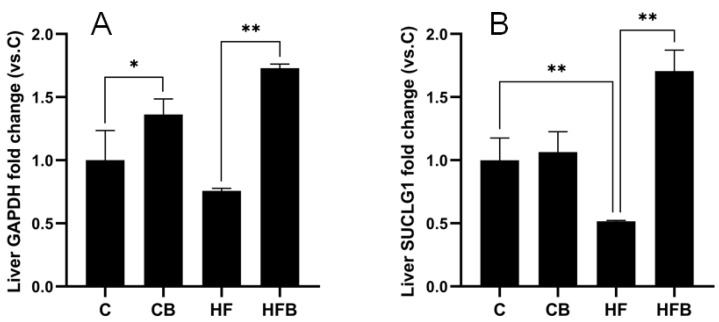
The expression of GAPDH (**A**) and SUCLG1 (**B**), as measured by MS protein profiling. The results are expressed as means ± SEM of the relative peak areas. * *p* < 0.05, ** *p* < 0.01.

**Figure 8:**
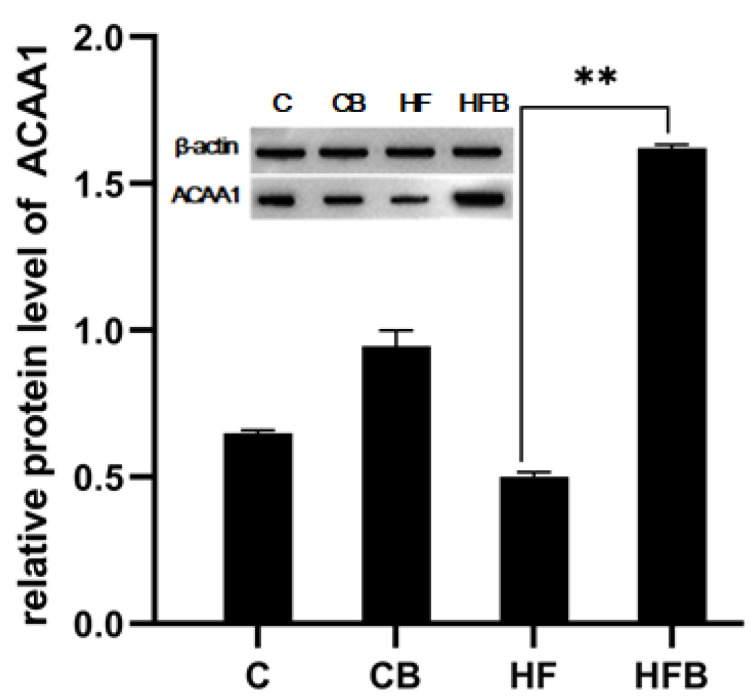
Verification of the expression of liver protein in the different groups. The Western blot bands of ACAA1A. Actin was used as reference. ** *p* < 0.01.

The results revealed significant differences in protein expression levels in murine liver among the C, CB, HF and HFB groups (Figure 6 and Figure 7). The addition of 1% betaine caused 4.71-fold protein expression of HADHA, 3.36-fold of ECHS1 and 13.99-fold of ACOX1, 3.72-fold of ACAT1 and 11.64-fold of ACAA1A in the mouse liver compared to the HF group (*p* < 0.01). However, the protein expression of APOA2 was only 87% of that of the HF group (*p* < 0.01) (Figure 6). As shown in Figure 7, the protein expression of SUCLG1 in the HF group was only 52% of that in the C group (*p* < 0.05). Meanwhile, the expression of GAPDH protein in the liver of mice in the HFB group was 2.29-fold and SUCLG1 protein expression was 3.31-fold than HF group (*p* < 0.01). Immediately following, Western blot analysis was used to verify the alteration of ACAA1 protein level. The results showed that the relative protein expression of ACCA1A in the liver of mice in the HFB group was 3.31 folds higher than that of mice in the HF group (Figure 8). In summary, these results revealed that the addition of 1% betaine affected the murine liver protein profiles response to an HF diet.

## 4. Discussion

The HF diet decreased hepatic homocysteine levels but increased plasma homocysteine, and it increased lipid metabolites and the NAD/NADH ratio, indicating that the HF diet induced fat accumulation via decreased β-oxidation resulting from abnormal lipid and Carbohydrate metabolism [20]. Dietary supplementation with methyl donors in adult rats can promote homocysteine transfer into methionine and reduce the concentration of homocysteine, thereby reducing liver fat accumulation in obesity-induced rats [21,22]. As an efficient methyl donor, betaine, an important participant in methionine metabolism, can promote the metabolism of fatty acids in the liver, thereby preventing non-alcoholic fatty liver disease caused by excessive accumulation of lipids [10,23]. Our results illustrated that in liver tissue, the HF diet decreased the expression of proteins involved in metabolic pathways, including fatty acid degradation, the TCA cycle and glycolysis pathway. Betaine supplementation alleviated the phenomenon of reduced metabolism of lipids and carbohydrates by upregulating the expression of proteins, including HADHA, ECHS1, ACOX1, ACAT1, ACAA1, APOA2, GAPDH and SUCLG1. We conjectured that the observed effects of betaine on reducing lipid accumulation in the liver may be explained by betaine increasing the expression of specific proteins in pathways associated with lipid metabolism and promoting the metabolism of fatty acids.

### 4.1. Direct Participation of Fatty Acid Oxidation Proteins in Lipid Metabolism

Fatty acid β-oxidation is carried out in mitochondria via carnitine, and obstruction of fatty acid oxidation leads to mitochondrial metabolism disorders [24]. ACOX1 is a marker of fatty acid metabolism and serves as a key enzyme for fatty acid β-oxidation and regulates fatty acid β-oxidation in the liver by regulating oxidative respiration in cells [25,26,27,28]. Research also showed that an HF diet induced lipid accumulation in the liver and hepatic steatosis in mice [29]. Another study confirmed that an HF diet significantly reduced the expression of the ACOX1 protein in the liver, which affected fat decomposition in rat liver cells [30]. In our study, 1% betaine in HF diet increased the expression of the ACOX1 protein, which might affect the fatty acid β-oxidation pathway by enhancing the activity of ACOX1 or increasing the rate of oxidative cellular respiration, and then promoted fatty acid oxidation in the liver.

ECHS1 is involved in the second step of the β-oxidation pathway, which involves the catalysis of mitochondrial fatty acids via the hydration of a 2-trans acyl CoA intermediate to L-3-hydroxy-CoAs [31]. Studies have shown that a reduction of ECHS1 might reduce triglyceride accumulation in the liver by attenuating fatty acid oxidation in the mitochondria via the PPAR pathway [32,33]. Another study showed that ECHS1 protein expression in broiler belly was upregulated, which promoted fatty acid oxidation and reduced abdominal fat deposition [34]. HF diet decreased the expression level of ECHS1, meanwhile the activity of fatty acid synthase is increased and accelerated the production of acetyl coenzyme, the precursor of fatty acid synthesis [35]. As expected, in the present study, 1% betaine supplementation significantly increased the expression of the ECHS1 protein, which accelerated the rate of fatty acid β-oxidation and reduced the deposition of abdominal fat. Furthermore, betaine supplementation reduced the body weights of the HF diet-induced mice.

Expression of the HADHA protein moderated the rate of fatty acid metabolism by catalyzing the last three steps of fatty acid beta-oxidation in liver mitochondria [36,37,38]. Reduction of HADHA protein expression leaded to an increase in triglyceride levels, and consequently to fatty liver [39]. Li analyzed the hepatic mitochondrial proteome of non-alcoholic steatohepatitis in rats fed an HF diet and found that the diet resulted in downregulation of HADHA protein expression in liver cells [40]. In our study, 1% betaine added to an HF diet increased HADHA protein expression in the liver. Based on this finding, we proposed that supplementation with betaine in an HF diet can help to combat HF diet-induced lipid accumulation in murine liver.

### 4.2. Participant of the PPARα Pathway in the Oxidation of Fatty Acid Proteins

Mitochondrial β-oxidation of fatty acids played an important role in lipid metabolism [41]. ACAT1 is the central enzyme for fatty acid β-oxidation in mitochondria, which is the only intracellular enzyme that catalyzes the formation of cholesteryl esters from free cholesterol and long-chain fatty acids [42,43,44]. Previous research demonstrated that the expression of the ACAT1 protein increased in response to elevated expression of the PPARα gene and protein [45].

ACAA1 is a target gene in the PPARα signaling pathway, which inhibits lipogenesis in the liver by both direct and indirect mechanisms [46]. ACAA1 protein expression is involved in fatty acid peroxisomal and mitochondrial oxidation in the liver [47]. A previous study reported that betaine upregulated PPARα and ameliorated lipid accumulation and insulin resistance in fructose-induced nonalcoholic fatty liver disease. In this study, betaine supplementation upregulated the protein expression of ACAT1 and ACAA1. These results support the idea that betaine prevented HF-diet induced obesity by regulating the PPARα pathway in murine liver.

APOA2 also functioned in the metabolism of free fatty acids and triglycerides [48], and it was the second most abundant apolipoprotein in the transport of high-density lipoproteins (HDL) [49]. Research has found that reduced HDL levels reduce bile salt synthesis [50], in addition to the PPARα signaling pathway activated by fatty acid β-oxidation prevents cholestatic liver injury [51]. Meanwhile, upregulation of APOA2 protein expression inhibited high density lipoprotein, mediated by PPARα [52]. In this study, HF diets increased the levels of triglycerides and free fatty acids in the liver, leading to an associated increase in APOA2 protein expression, however the HFB group showed a highly significant decrease in APOA2 protein expression. So, we concluded that betaine supplementation in an HF diet might inhibit APOA2 expression, mediated by the PPARα pathway.

### 4.3. Participant of the Acceleration of Carbohydrate Metabolism

SUCLG1 is an important catalytic enzyme in the TCA cycle. SUCLG1 is an α-subunit and mitochondrial matrix enzyme, which converts guanosine diphosphate to guanosine triphosphate or adenosine diphosphate (ADP) to adenosine triphosphate (ATP) and converts succinyl-CoA to succinate and free CoA [39,53], thereby inhibiting the activity of the TCA pathway and increasing fatty acid metabolism. In the present study, the results of the liver proteomic analysis showed that SUCLG1 was downregulated in the HF diet group. In addition, 1% betaine supplementation in the HF diet increased the expression of the SUCLG1 protein compared with that in the HF group. We assume that the possible reason for this was the acceleration of the TCA cycle owing to the increased consumption of ATP, thereby causing an increase in the protein expression level of SUCLG1.

In glycolysis, GAPDH induces the phosphorylation of glyceraldehyde-3-phosphate into 1,3-bisphosphoglycerate [54]. A decrease in GAPDH protein expression results in an accumulation of glycolytic intermediate in the glycolytic pathway and blocks the intracellular glycolytic pathway [55], and ultimately triggered cellular oxidative stress and DNA damage [56]. Another study found that the expression of the GAPDH protein was decreased in the livers of mice fed an HF diet [57]. The results of our study suggested that GAPDH protein expression decreased in the HF group and that betaine increased GAPDH protein expression, thereby promoting glycolysis in liver.

## 5. Conclusions

In summary, supplementation with 1% betaine enhanced fatty acid metabolism in the liver of HF diet mice by increasing protein expression levels of key enzymes involved in fatty acid β-oxidation and regulating the activity of the PPAR signaling pathway. In addition, betaine elevated protein expression of catalytic enzymes involved in the TCA cycle and glycolytic process. In the present study, the effects of betaine on the liver protein profile of mice on HF diet were analyzed to reveal the effects of betaine on the pathways related to lipid synthesis and metabolism and the activities of key biochemical reaction-related enzymes in the liver of mice on HF diet, yet the results of this study were not validated in fatty liver cellular experiments, and similar studies in future research may be conducted on fatty liver cellular models and more insightful mechanisms of effects will be discovered to supplement the existing findings on betaine in fatty liver.

## Figures and Tables

**Table 1:** Details of the diets. The control diet was a conventional D12450B diet. More than 20% energy added to HF diet.

	Control		HF	
Ingredients	g	kcal	g	kcal
Casein, 80 Mesh	200	800	200	800
L-cysteine	3	12	3	12
Com starch	315	1260	72.8	291
Maltodextrin 10	35	140	100	400
Sucrose	350	1400	172.8	691
Cellulose, BW200	50	0	50	0
Soybean oil	25	225	25	225
Lard	20	180	177.5	1598
Mineral mix S10026	10	0	10	0
Dicalcium phosphate	13	0	13	0
Calcium carbonate	5.5	0	5.5	0
Potassium citrate, 1 H_2_O	16.5	0	16.5	0
Vitamin Mix V10001	10	40	10	40
Choline bitartrate	2	0	2	0
Total	1055	4057	858.1	4057

## Data Availability

Not applicable.
